# Protective efficacy of a universal influenza mRNA vaccine against the challenge of H1 and H5 influenza A viruses in mice

**DOI:** 10.1002/mlf2.12085

**Published:** 2023-09-24

**Authors:** Yulei Li, Xi Wang, Xi Zeng, Wenbo Ren, Pu Liao, Baoli Zhu

**Affiliations:** ^1^ Savaid Medical School University of Chinese Academy of Sciences Beijing China; ^2^ The Key Laboratory of Molecular Pathology (Hepatobiliary Diseases) of Guangxi, Department of Pathology The Affiliated Hospital of Youjiang Medical University for Nationalities Baise China; ^3^ CAS Key Laboratory of Pathogenic Microbiology and Immunology Chinese Academy of Sciences Beijing China; ^4^ Beijing Children's Hospital Capital Medical University Beijing China; ^5^ College of Life Sciences Jiangxi Science and Technology Normal University Nanchang China; ^6^ Department of Clinical Laboratory Chongqing General Hospital Chongqing China; ^7^ University of Chinese Academy of Sciences Beijing China; ^8^ Department of Pathogenic Biology, School of Basic Medical Sciences Southwest Medical University Luzhou China

**Keywords:** immunogenicity, influenza virus, mRNA vaccine, universal vaccine

## Abstract

Current influenza vaccines need to be updated annually owing to constant antigenic drift in the globular head of the viral surface hemagglutinin (HA) glycoprotein. The immunogenic subdominant stem domain of HA is highly conserved and can be recognized by antibodies capable of binding multiple HA subtypes. Therefore, the HA stem antigen is a promising target for the design of universal influenza vaccines. On the basis of an established lipid nanoparticle‐encapsulated mRNA vaccine platform, we designed and developed a novel universal influenza mRNA vaccine (mHAs) encoding the HA stem antigen of the influenza A (H1N1) virus. We tested the efficacy of the mHAs vaccine using a mouse model. The vaccine induced robust humoral and specific cellular immune responses against the stem region of HA. Importantly, two doses of the mHAs vaccine fully protected mice from lethal challenges of the heterologous H1N1 and heterosubtypic H5N8 influenza viruses. Vaccinated mice had less pathological lung damage and lower viral titers than control mice. These results suggest that an mRNA vaccine using the conserved stem region of HA may provide effective protection against seasonal and other possible influenza variants.

## Impact statement

Influenza is a global health problem. Seasonal influenza vaccines have limited effectiveness. A universal influenza virus vaccine that elicits broad immune responses against different types of influenza viruses is necessary to reduce the incidence of influenza. Here, we describe a novel universal influenza mRNA vaccine based on the stem region of the hemagglutinin (HA) that induced strong specific humoral and cellular immune responses in mice and protected them against challenges with lethal doses of H1N1 and H5N8 influenza viruses. Our results suggest that an mRNA vaccine using the conserved stem region of HA can be used as a candidate for a universal influenza virus vaccine.

## INTRODUCTION

Influenza virus infection remains a serious threat to global health and the world economy^1^. According to the World Health Organization, annual influenza epidemics cause an estimated three to five million cases of severe illness and 290,000–650,000 deaths worldwide each year^2^. The influenza virus is a segmented, negative single‐stranded RNA virus. The influenza virus is prone to gene reassortment and antigenic drift because of its unique gene structure and antigenic characteristics. Therefore, this virus escapes previous natural immunity, resulting in low effectiveness of seasonal influenza vaccines, ranging from 10% to 60%^3^. Consequently, the development of a highly effective universal influenza vaccine that can provide broad and effective protection against a wide range of influenza viruses is essential.

An effective universal influenza vaccine design strategy uses the highly conserved stem domain of the hemagglutinin (HA) glycoprotein as an immunogen^4^. Recent studies have shown that monoclonal antibodies isolated from mice and humans targeting this domain broadly neutralize multiple influenza virus strains in vitro and protect animals against an influenza virus challenge^5^, ^6^, ^7^. Headless H1 stem region‐stabilized protein nanoparticle vaccines preserve the natural conformation of the HA stem and effectively protect against homologous H1N1 and heterosubtypic H5N1 virus infections^8^. However, the production of these subunit vaccines requires complex protein purification processes and effective and safe adjuvants. Therefore, a better vaccine platform for the development of new vaccine types is required.

mRNA is an attractive vaccine platform, with the advantages of safety, rapid development, potent immunogenicity, simple vaccine design, and ease of manufacture compared with traditional vaccine platforms. The coronavirus disease 2019 (COVID‐19) pandemic has shown the enormous potential of mRNA vaccines against severe acute respiratory syndrome coronavirus 2 infection in humans. This success has stimulated the development of mRNA–lipid nanoparticle (LNP) vaccines against other pathogens including the influenza virus. In this study, we report the efficacy and immunogenicity of a universal influenza mRNA vaccine based on the conserved HA stem region. This vaccine showed strong specific humoral and cellular immune responses and provided robust protection against heterologous H1N1 and heterosubtypic H5N8 viruses in mice. The properties of the universal influenza mRNA platform make it amenable for rapid scale‐up, which is vital during pandemics.

## RESULTS

### Construction and characterization of the mRNA vaccine

**Figure 1:**
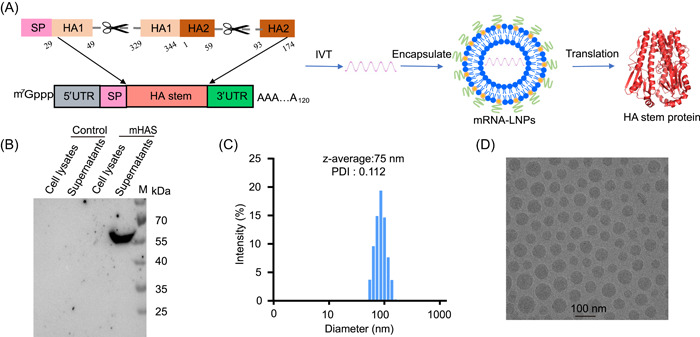
Construction and characterization of the mHAs vaccine. (A) Schematic diagram of mHAs mRNA construction. The synthesized nucleoside‐modified mHAs mRNA was encapsulated with LNPs to form mRNA–LNPs. LNP, lipid nanoparticle; SP, signal peptide; UTR, untranslated region. (B) Protein mHAs expression in the cell lysate and supernatant after the mHAs mRNA was transfected into HEK293T cells at 48 h was analyzed using Western blot analysis. (C) Representative intensity graph of the mHAs vaccine measured using a dynamic light‐scattering method. PDI, polydispersity index. (D) A representative cryo‐electron microscopic image of mHAs solution following mRNA encapsulation. Scale bar, 100 nm.

We designed an mRNA vaccine that encoded the conserved stem domain of the HA glycoprotein of A/Victoria/2570/2019 (H1N1). The amino acid (aa) residues of the HA1 (aas 29–49 and 329–344) and HA2 (aas 1–59 and 93–174) domains were included as vaccine targets (Figures 1A and S1). The codons and sequences in the mRNA construct were optimized using the proprietary GenScript algorithm to increase the translation of the encoded protein. We introduced point mutations in the hydrophobic patches of the HA2 stem domain (K51M, Y94D, N95L, and E103L) (Figure S1). These point mutations stabilize the inner core of the HA stem HA2, weaken strong hydrophobic interactions, and avoid protein aggregation in neutral pH conformation^8^, ^9^, ^10^. Before the vaccination experiments, mRNA protein expression was confirmed by cell transfection in vitro. This experiment showed that HA stem protein was produced effectively and secreted into the supernatant of HEK293T cells (Figure 1B). The mHAs–LNP formulations were prepared using a modified procedure for siRNA, as described previously^11^. We used the RiboGreen fluorescence assay (Molecular Probes/Invitrogen) to confirm that the encapsulation efficacy of mHAs was >95%. Dynamic light scattering of the mHAs vaccine had an average particle size of 75 nm, and the polydispersity index was 0.112 (Figure 1C). Cryo‐electron microscopy showed that mHAs particles were homogeneous spheroids with an electron‐dense core (Figure 1D).

### Humoral immune responses elicited by mHAs vaccination

**Figure 2:**
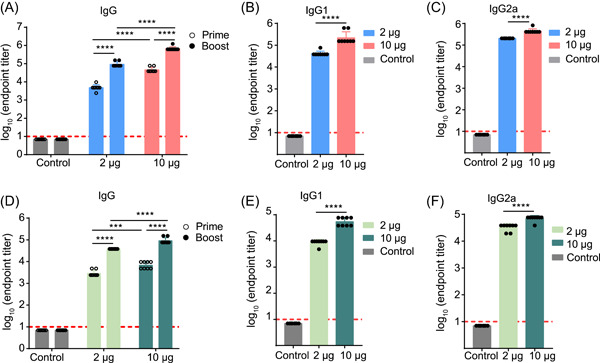
Immunogenicity of the mHAs vaccine in mice tested at 19 and 35 days after the initial vaccination. (A–C): Measurement of the H1‐specific IgG (A), IgG1 (B), and IgG2a (C) endpoint geometric mean titers elicited by the mHAs vaccine. (D–F): Measurement of the H5‐specific IgG (D), IgG1 (E), and IgG2a (F) endpoint geometric mean titers elicited by the mHAs vaccine. ***p < 0.001 and ****p < 0.0001.

To evaluate the humoral immunity of the mHAs vaccine, female BALB/c mice (*n* = 8) were vaccinated twice, 3 weeks apart, with a low dose (2 μg) or a high dose (10 μg) of the mHAs vaccine. Empty LNPs were used as a control. Serum samples collected 19 and 35 days after the initial vaccination were evaluated for H1‐ and H5‐specific immunoglobulin G (IgG) and IgG subtypes using an enzyme‐linked immunosorbent assay (ELISA). The mHAs vaccine elicited a strong H1‐ and H5‐specific total IgG response after the prime and boost vaccinations in all mice (Figure 2A,D). Boost vaccination with mHAs resulted in a rapid increase in IgG antibodies. The endpoint geometric mean titers (GMTs) of H1‐specific IgG in mice vaccinated with the 10‐μg dose were greater than 10^5^ and significantly higher than those in mice vaccinated with the 2‐μg dose. However, IgG antibodies were detected in the serum samples of mice vaccinated with the control (Figure 2A). Following the boost dose, mice vaccinated with 10 μg of mHAs showed cross‐reactive H5‐specific IgG with GMTs >10^4^ (Figure 2D). Furthermore, both doses elicited IgG2a and IgG1 subclass H1‐ and H5‐specific antibodies (Figure 2B–F). The T helper 1 (T_H_1) antibody (IgG2a) titers were higher than the T_H_2 antibody (IgG1) titers, which suggested that the mHAs vaccine induced a T_H_1‐biased antibody response.

### Cellular immune responses elicited by mHAs vaccination

**Figure 3:**
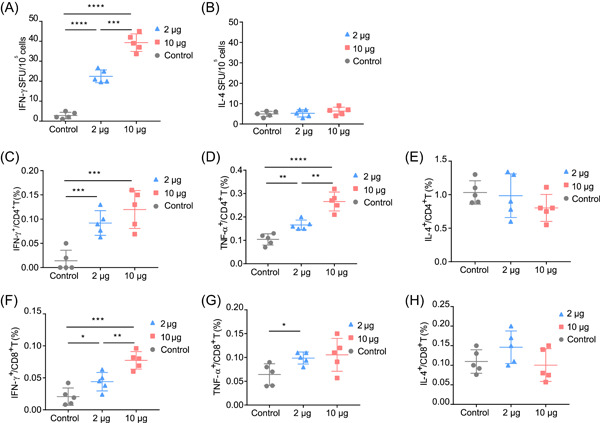
Cellular immune responses elicited by the mHAs vaccine in mice tested at 35 days after the initial vaccination. An ELISPOT assay was performed to evaluate the capacity of splenocytes to secrete IFN‐γ (A) and IL‐4 (B) following restimulation with H1 stalk peptide pools. FACS analysis results showing the percentages of CD4^+^ T cells secreting IFN‐γ (C), TNF‐α (D), or IL‐4 (E) after stimulation. Empty LNPs were used as the control. FACS analysis results showing the percentages of CD8^+^ T cells secreting IFN‐γ (F), TNF‐α (G), or IL‐4 (H) after stimulation with H1 peptide pools. ELISPOT, enzyme‐linked immunospot; FACS, fluorescence‐activated cell sorting; IFN‐γ, interferon‐γ; IL‐4, interleukin‐4; LNP, lipid nanoparticle; TNF‐α, tumor necrosis factor‐α. *p < 0.05, **p < 0.01, ***p < 0.001, and ****p < 0.0001.

We further characterized the cellular immune response induced by the mHAs vaccine and performed enzyme‐linked immunospot (ELISPOT) and intracellular cytokine staining assays. The spleens of BALB/c mice (*n* = 5) were harvested 35 days after the initial vaccination. After restimulation with H1 stalk peptide pools in vitro, the mean (±SEM) interferon‐γ (IFN‐γ) spot‐forming units per million splenocytes in mice vaccinated with 2 and 10 μg of the mHAs vaccine were significantly higher than those in mice vaccinated with the control (22.5 ± 1.4 and 39 ± 1.9, respectively, vs. 2.8 ± 0.7). However, few interleukin‐4 (IL‐4) spot‐forming units above the plate were detected (Figure 3A,B). Cytokine‐producing CD4^+^ and CD8^+^ T cells in splenocytes were analyzed using flow cytometry with ICS. The percentage of CD4^+^ T cells secreting IFN‐γ and tumor necrosis factor‐α (TNF‐α) was high in mice vaccinated with 2 and 10 μg of the mHAs vaccine, while the percentage of cells secreting IL‐4 was low (Figure 3C–E). In CD8^+^ T cells, the percentage of cells secreting IFN‐γ and TNF‐α was also significantly increased in mice vaccinated with 2 and 10 μg of the mHAs vaccine, while there was no significant difference in IL‐4‐secreting cells compared with the control vaccination (Figure 3F–H). These results suggest that the mHAs vaccine effectively induces a T_H_1‐biased, HA stem‐specific cellular immune response.

### Efficacy of the mHAs vaccine in protecting mice from a heterologous H1N1 challenge

**Figure 4:**
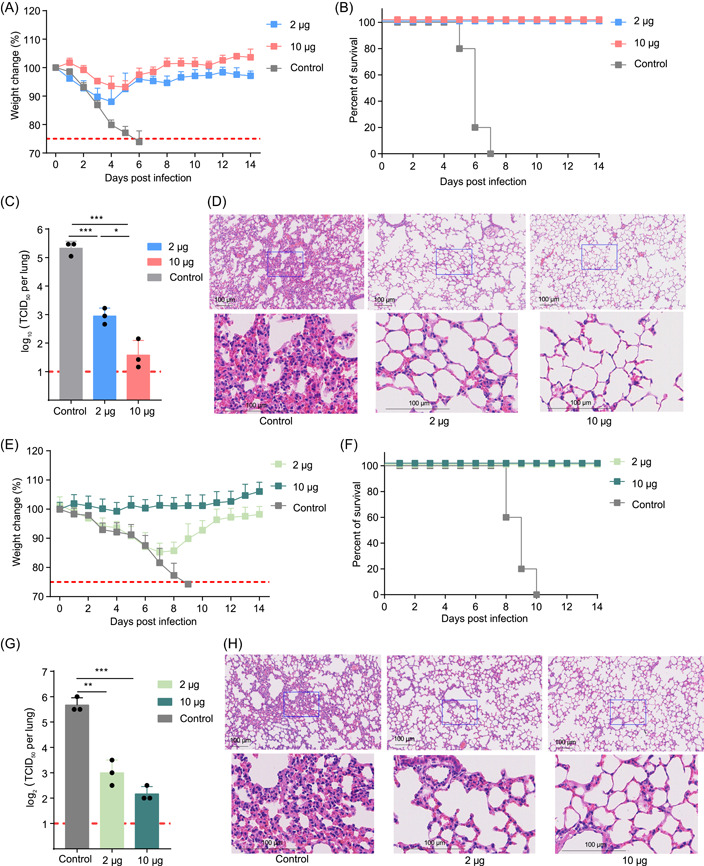
Protective efficacy of the mHAs vaccine against a challenge with the heterologous H1N1 and heterosubtypic H5N8 influenza viruses. Groups of 6–8‐week‐old female BALB/c mice (*n* = 8) were vaccinated with 2 or 10 μg of mHAs vaccine at 3‐week intervals. The mice were intranasally challenged with 20 × LD_50_ of the A/Brisbane/02/2018 (H1N1) virus. Lung tissue was harvested and split for detecting the viral titer and a pathological examination (*n* = 3). Vaccine efficacy was assessed by measuring morbidity (weight loss) (A), mortality (survival) (B), lung viral titers (C) on Day 3 postchallenge, and histological pathological analyses (D). Groups of 6–8‐week‐old female BALB/c mice (*n* = 8) were vaccinated with 2 or 10 μg of mHAs vaccine at 3‐week intervals. The mice were intranasally challenged with 10 × LD_50_ of the A/Astrakhan/3212/2020 (H5N8) virus reassortment. Lung tissue was harvested and split for detecting the viral titer and a pathological examination (*n* = 3). Vaccine efficacy was assessed by measuring morbidity (weight loss) (E), mortality (survival) (F), lung viral titers (G) on Day 3 postchallenge, and histopathology (H). LD_50_, lethal dose 50%; TCID_50_, 50% tissue culture infective dose. *p < 0.05, **p < 0.01, and ***p < 0.001.

To examine the protective potential of the mHAs vaccine against the H1N1 influenza virus, BALB/c mice (*n* = 8) were vaccinated twice, with a low dose (2 μg) or a high dose (10 μg) of the mHAs vaccine. Two weeks after the boost vaccination, the vaccinated mice were intranasally challenged with 20× lethal dose 50% (LD_50_) of the A/Brisbane/02/2018 (H1N1) virus. Three mice were euthanized and necropsied 3 days postinfection, and their lungs were collected for quantifying the infectious H1N1 virus in lung tissue homogenates. We found that the mHAs‐vaccinated group had a high efficacy of protection against lethal H1N1 infection as shown by less weight loss (approximately 10%) and a 100% survival rate, whereas all of the mice in the control group died within 6 days (Figure 4A,B). Moreover, vaccination with the mHAs vaccine significantly reduced the viral load in the lung tissue of mice (Figure 4C). Histopathology showed that the control mice had more extensive and severe lung damage, with consolidated lesions and inflammatory cell infiltration, than mice administered the mHAs vaccine. In contrast, the lungs of mice vaccinated with the mHAs vaccine only showed slight histological changes (Figure 4D).

### Efficacy of the mHAs vaccine in protecting mice from a heterosubtypic H5N8 challenge

We next assessed whether the mHAs vaccine could protect mice against a lethal challenge with the heterosubtypic H5N8 influenza virus. BALB/c mice (*n* = 8) were vaccinated using the same two‐dose vaccination regimen as described above. Two weeks after the boost vaccination, vaccinated mice were intranasally challenged with 10 × LD_50_ of the A/Astrakhan/3212/2020 (H5N8) virus reassortment. Nine days following the H5N8 challenge, all mice vaccinated with the control reached humane endpoints and were euthanized. The mHAs vaccine provided 100% protection against mortality in mice (Figure 4E,F). Consistently, the viral load in the lungs of mice vaccinated with the mHAs vaccine was significantly lower than that in mice vaccinated with the control (Figure 4G). A histopathological examination showed severe bronchopneumonia and interstitial pneumonia in mice vaccinated with the control, whereas only minor bronchopneumonia was observed in mice vaccinated with the mHAs vaccine (Figure 4H). These results indicate that the mHAs vaccine can effectively protect mice against heterosubtypic H5N8.

## DISCUSSION

The high diversity of influenza viruses poses a challenge to development of effective influenza vaccines. Enhancing the cross‐reactivity of the immune response is important to develop a universal influenza vaccine. Previous studies have identified several viral protein regions that are conserved in different subtypes of influenza viruses, including epitopes in the stem domain of HA, neuraminidase, the ectodomain of the ion channel M2, nucleoprotein, and matrix protein^12^. Vaccines that target these regions may induce a wide range of protective influenza virus‐specific immune responses. Among the most widely studied targets is the subdominant HA stem^13^. In most individuals, the seasonal influenza vaccine shows a poor antibody response to the HA stem^14^, ^15^. Therefore, the development of new vaccine platforms and optimization of the immunogen to specifically trigger antibodies against this region are important. Several approaches have been explored in the process of developing a universal influenza vaccine that would elicit cross‐reactive antibody responses to the influenza HA stem. These approaches include the development of “headless” HA constructs^8^, ^9^, hyperglycosylation of the head domain^16^, and the development of a chimeric (prime‐boost) or mosaic HA stem via sequential vaccination with “exotic” heads^17^, ^18^, ^19^, ^20^. Impagliazzo et al.^9^ designed headless H1 miniHA stem proteins with various monomers, dimers, and trimers, and found that trimer construction as a vaccine better protected mice from an H5N1 virus challenge. Yassine et al.^8^ used a structure‐based design to remove the immunodominant domain in the HA head domain. Using a nanoparticle antigen display platform, they constructed an HA stem nanoparticle vaccine immunogen and induced protective immunity against a heterosubtypic H5N1 virus in mice and ferrets. However, cumbersome protein expression and purification, and the multistep preparation of nanoparticles may pose concerns regarding scaling up vaccine production and the chemical modification of potential epitopes.

In this study, we developed a nucleoside‐modified novel universal influenza mRNA vaccine named mHAs, which expresses HA stem regions on entry into cells. After two doses of this vaccine, mice vaccinated with the 2‐ or 10‐µg dose had high titers of H1‐ and H5‐specific antibodies. Additionally, this vaccine provided effective protection against challenges with lethal doses of the heterologous A/Brisbane/02/2018 (H1N1) virus and the heterosubtypic A/Astrakhan/3212/2020 (H5N8) virus. Furthermore, vaccinated mice showed a significant decrease in the viral load and pathological lung damage. There are several possible immune mechanisms for broad protection by HA stem vaccination. There is growing evidence that antibodies against the HA stem can induce effective in vivo protection, which is largely mediated by antibody‐dependent cellular cytotoxicity and is highly dependent on Fc–FcγR interactions^21^, ^22^, ^23^. In line with this evidence, a recent evaluation of an mRNA vaccine containing four conserved antigens of influenza A (HA stem, neuraminidase, M2, and nucleoprotein) showed that specific antibodies against the HA stem had no neutralizing activity, but had strong antibody‐dependent cellular cytotoxicity effects^24^. IgG subclasses play a critical role in influenza vaccine efficacy because of their relative affinities for activating and inhibitory FcγRs, and thus their different effector capabilities. IgG2a monoclonal antibodies against stem HA are more effective in protecting against lethal influenza virus infection than IgG1 antibodies with the same epitope specificity^22^. In this study, all mice vaccinated with the mHAs vaccine produced high titers of IgG1 and IgG2a subtypes, with higher titers of IgG2a than IgG1. This finding suggests that antibodies induced by the mHAs vaccine are effective at clearing the virus.

T cells elicit protection against pandemic influenza strains^25^, ^26^. Our vaccination experiments showed that the proportion of spleen cells that secreted IFN‐γ and those of CD4^+^ and CD8^+^ cells that secreted IFN‐γ and TNF‐α were significantly increased in mice vaccinated with the mHAs vaccine after restimulation with multiple peptides from H1N1. This finding suggests that the vaccine can induce a strong T_H_1‐directed immune response, consistent with the observations of other mRNA vaccines^27^, ^28^, ^29^. Cell‐mediated immunity plays an important role in preventing influenza, heterotypic immunity, and establishing memory. Therefore, further evaluation of the role of T cells in mediating broad protective immunity, as observed in recent influenza mRNA vaccine studies^30^, ^31^, would be interesting.

Nucleoside‐modified mRNA‐LNP vaccines have emerged as an attractive vaccine platform for the control of infectious diseases^32^, ^33^. The mRNA vaccines are produced through a well‐controlled, enzymatic, and rapidly scalable process that is independent of the sequence produced and can be used for almost any pathogen. In addition, host cell production and presentation of immunogens are more similar to the process of viral infection than exogenously produced, purified, and formulated protein antigens. These immunogens have advantages of high speed, accuracy, and adaptability of antigen design and production control that cannot be replicated using traditional platforms. These features are particularly important for emerging infectious diseases like potential pandemic influenza. Our data highlight the potential of an mRNA vaccine expressing conserved influenza antigens as an effective vaccine against seasonal and other possible influenza variants.

## MATERIALS AND METHODS

### Cells, viruses, and animals

HEK293T cells (ATCC) and Madin–Darby Canine Kidney (MDCK) cells (ATCC) were cultured at 37°C in Dulbecco's modified Eagle's medium (DMEM) supplemented with 100 U/ml penicillin, 100 µg/ml streptomycin, and 10% fetal bovine serum. A wild‐type A/Brisbane/02/2018 (H1N1) virus was used as a heterologous H1N1 challenge virus. The heterosubtypic H5N8 challenge virus was a 6:2 reassortment virus of A/Astrakhan/3212/2020 (H5N8), in which HA and neuraminidase were from A/Astrakhan/3212/2020 (H5N8), in an A/Puerto Rico/8/1934 backbone. BALB/c mice were purchased from Beijing Vital River Animal Technology Co., Ltd. (licensed to Charles River Laboratories). All mice were provided free access to water and standard rat food, and were housed under a 12‐h, light–dark cycle (temperature: 20–25°C, humidity: 40%–70%). All mice used in this study were healthy and were not used in other experimental procedures.

### Design and synthesis of nucleoside‐modified mHAs mRNA

The mHAs mRNA with modified nucleosides was constructed and synthesized as follows: Briefly, the HA gene sequence of the influenza A virus A/Victoria/2570/2019 (H1N1) was acquired from GISAID (ID: EPI1801581) and used to design the H1 stem vaccine construct. The aa residues of the HA1 (aas 29–49 and 329–344) and HA2 (aas 1–59 and 93–174) domains were included as a vaccine target based on the major conserved region of the HA stem and stabilizing domaindo^8^, ^9^. The target gene was synthesized (Genscript Biotech Co., Ltd.) and linked to the constructed pUC57 vector with a T7 promoter. The plasmid was linearized by enzyme digestion and in vitro transcription using a T7 RNA polymerase kit (Novoprotein Biotech Co., Ltd.). Additionally, 1‐methylpseudourine‐5′‐triphosphate was used instead of UTP to generate modified nucleoside‐containing mRNA. Capped mRNA was produced using a Cap1 Capping System kit (Novoprotein). The mRNA was purified by overnight 7.5 M lithium chloride precipitation at −20°C. The precipitate was pelleted by centrifugation at 12,000 rpm for 15 min at 4°C, washed with 70% ethanol, centrifuged at 12,000 rpm for 5 min at 4°C, and dissolved in RNase‐free water. Purified mRNA was analyzed using agarose gel electrophoresis and stored frozen at −80°C until use.

### Antigen expression

HEK 293T cells, which were pre‐plated in a 12‐well plate, were transfected with mRNA encoding immunogen using Lipofectamine 3000 Transfection Reagent (Thermo Fisher Scientific) according to the manufacturer's instructions. To facilitate the detection of target antigen expression, a flag label was added to the C‐terminal of the target antigen. The supernatant was collected, and cells were lysed at 48 h after transfection. The expression of antigen proteins was analyzed by Western blot.

### LNP formulation of mRNA

The mRNA was encapsulated in LNPs using a self‐assembly process. Lipids were dissolved in ethanol at a molar ratio of 50:10:38.5:1.5 ((6Z,9Z,28Z,31Z)‐heptatriacont‐6,9,28,31‐tetraene‐19‐yl4‐(dimethylamino)butanoate:1,2‐distearoyl‐sn‐glycero‐3‐phosphocholine:cholesterol:polyethylene glycollipid). The lipid mixture was combined with 50 mM citrate buffer (pH 4.0) containing mRNA at a ratio of 1:3 (ethanol:aqueous) using a microfluidic mixer (Precision Nanosystems) at a flow rate of 12 ml/min. The mRNA was stored at 4°C at a concentration of RNA of approximately 1 mg/ml.

### Cryo‐electron microscopy of LNPs

The mRNA–LNP sample was transferred onto a glow‐discharged ultrathin carbon‐coated copper grid (Beijing Zhongjingkeyi Technology Co., Ltd.), which was vitrificated using Vitrobot Mark IV (Thermo Fisher Scientific). The frozen grids were loaded into a Talos 120c transmission electron microscope (Thermo Fisher Scientific). Sample images were produced using a direct electron detector (ED20) at a total electron dose of approximately 50e−/Å^2^.

### Mouse vaccination and challenge experiments

On Days 0 and 21, 6–8‐week‐old female BALB/c mice were vaccinated intramuscularly with mHAs mRNA‐LNP (2 μg/100 μl/mouse and 10 μg/100 μl/mouse) or empty LNPs (control). Serum samples were collected at specified time points after vaccination and tested for specific antibody responses, as described below. Spleen tissue was collected at Day 35 postvaccination to evaluate the cellular immune responses using an ELISPOT assay and flow cytometry, as described below.

Two weeks after the boost, the mice were challenged with a lethal dose of influenza A/Brisbane/02/2018 (H1N1) (20 × LD_50_) and A/Astrakhan/3212/2020 (H5N8) (10 × LD_50_). After this challenge, the mice were monitored for 14 days to record changes in body weight and survival rates. A weight loss of >25% was considered as the humane endpoint. To enable viral load analysis, three mice were euthanized 3 days after challenge, the left lobe of the lung was collected, and the right lung was used for a histopathological analysis.

### ELISA

H1‐ or H5‐specific antibodies of different subtypes (IgG, IgG1, and IgG2a) were measured in serum samples using an ELISA. In brief, ELISA plates were coated overnight with 2 μg/ml of H1 or H5 recombinant protein, blocked with 5% fat‐free milk in phosphate‐buffered saline, and then incubated with serially diluted murine serum. The plates were incubated for 1 h at 37°C and washed. The plates were then incubated with goat antimouse IgG‐HRP (1:5000), IgG1‐HRP (1:5000), or IgG2a‐HRP (1:5000) antibodies (Abcam) for 1 h at 37°C and washed. The plates were then developed with the 3,3′,5,5′‐tetramethylbenzidine substrate. The reactions were stopped with 2 M H_2_SO_4_, and the absorbance was measured at 450 nm using a microplate reader (PerkinElmer). The endpoint titers were defined as the highest reciprocal dilution of serum to provide an absorbance >2.5‐fold of the background values. Antibody titers below the limit of detection were regarded as half the value at the limit of detection.

### ELISPOT assay

ELISPOT assay cellular immune responses in vaccinated mice were assessed using IFN‐γ or IL‐4 precoated ELISPOT kits (Dakewe Biotech Co., Ltd.) according to the manufacturer's protocol. Briefly, single‐cell suspensions obtained from mouse spleens (1 × 10^5^ cells/well) were ground in cell strainers and seeded onto precoated ELISPOT plates. Subsequently, the cells were incubated with peptide pools (15‐mers overlapping by 13 aas, 2 µg/ml each) covering A/Victoria/2570/2019 (H1N1) HA stem proteins (GenScript) and cultured at 37°C with 5% CO_2_ for 20 h. The same volume of 40% dimethyl sulfoxide (Sigma‐Aldrich), which was used to dissolve the peptide pool, was used as the negative control. Phorbol 12‐myristate 13‐acetate/ionomycin (Dakewe Biotech) was used as the positive control. The spots were counted and analyzed using CTL‐ImmunoSpot S5 (Cellular Technology Limited).

### Flow cytometric analysis of intracellular cytokines

CD4^+^ and CD8^+^ T‐cell responses were evaluated using ICS. Briefly, mouse splenocytes were added to a cell plate (1 × 10^6^ cells/well) and then stimulated with the peptide pool (2 μg/ml of individual peptides) for 4 h. Dimethyl sulfoxide (40%) and phorbol 12‐myristate 13‐acetate/ionomycin were used as negative and positive controls, respectively. The cells were incubated with GolgiPlug (BD Biosciences) for an additional 12 h at 37°C. The cells were washed with phosphate‐buffered saline (PBS) and surface‐stained with anti‐mouse CD3/CD4/CD8 antibodies (BioLegend) for 30 min at room temperature. The cells were subsequently fixed, permeabilized in permeabilizing buffer (BD Biosciences), and stained with fluorochrome‐labeled antimouse IFN‐γ/TNF‐α/IL‐4antibodies (BioLegend). An LSRFortessa flow cytometer (BD Biosciences) was used to acquire the flow cytometry data, which were then analyzed using FlowJo software (Becton Dickinson).

### Determination of the lung viral load

The viral load in lung homogenates was measured using a 50% tissue culture infective dose (TCID_50_) assay and MDCK cells. Briefly, MDCK cells were seeded into 96‐well flat‐bottom plates and incubated overnight (37°C, 5% CO_2_). The cells were washed with PBS and then incubated for 48 h at 37°C with serial dilutions of the lung homogenates in quadruplicate in DMEM supplemented with 100 U/ml penicillin, 100 µg/ml streptomycin, and 2 μg/ml of l‐1‐tosylamido‐2‐phenylethyl chloromethyl ketone‐treated trypsin. The TCID_50_ values were calculated using the Reed–Muench method.

### Histopathology assay

Mouse lung tissue was fixed in 4% neutral‐buffered formalin for 48 h, embedded in paraffin, and stained with hematoxylin and eosin. Images were captured using a LEICA Versa 200 Slide Autoloader (Leica Biosystems Imaging Inc.) and processed using K‐Viewer 1.5.5.8 Digital Slide Reading Software (Ningbo Konfoong Bioinformation Tech Co., Ltd.).

### Statistical analysis

All data plotted with error bars are expressed as the mean with standard deviation, unless otherwise indicated. Two‐tailed *p* values were calculated using the independent‐samples *t*‐test with Prism 7 (GraphPad Software).

## AUTHOR CONTRIBUTIONS

**Yulei Li**: Conceptualization (supporting); data curation (lead); formal analysis (lead); investigation (lead); methodology (lead); project administration (supporting); software (lead); validation (supporting); visualization (lead); writing—original draft (lead); writing—review and editing (supporting). **Xi Wang**: Data curation (supporting); investigation (supporting); methodology (supporting). **Xi Zeng**: Investigation (supporting); validation (supporting). **Wenbo Ren**: Investigation (supporting); validation (supporting). **Pu Liao**: Conceptualization (supporting); funding acquisition (supporting); supervision (supporting); writing—review and editing (supporting). **Baoli Zhu**: Conceptualization (lead); formal analysis (lead); funding acquisition (lead); methodology (lead); project administration (lead); resources (lead); supervision (lead); writing—review and editing (lead).

## ETHICS STATEMENT

This study was performed in strict accordance with the recommendations described in the Guide for the Care and Use of Laboratory Animals of the Institute of Microbiology, Chinese Academy of Sciences (IMCAS) Ethics Committee. All of the animal experiments were reviewed and approved by the Committee on the Ethics of Animal Experiments of IMCAS.

## CONFLICT OF INTERESTS

The authors declare no conflict of interests.

## Supporting information


**Figure S1.** Immunogen sequence alignment and design. HA gene sequence alignment of mHAs, A/Victoria/2570/2019(H1N1), and A/Astrakhan/3212/2020(H5N8). Amino acid sites with identical sequences are shown in red, mHA mutation sites are shown in green, and linking peptides are shown in blue. Deletions are represented by dots.

## Data Availability

All data supporting the findings of this study are available within the article or from the corresponding author upon reasonable request.
