# Supplementary material for: Protective efficacy of a universal influenza mRNA vaccine against the challenge of H1 and H5 influenza A viruses in mice
Source: mLife. 2023 Sep 24;2(3):308–16. doi: 10.1002/mlf2.12085 (PMC10989953; doi:10.1002/mlf2.12085)
Supplement: Supplementary file 1 — Figure S1. Immunogen sequence alignment and design. HA gene sequence alignment of mHAs, A/Victoria/2570/2019(H1N1), and A/Astrakhan/3212/2020(H5N8). Amino acid sites with identical sequences are shown in red, mHA mutation sites are shown in green, and linking peptides are shown in blue. Deletions are represented by dots. [file MLF2-2-308-s001.pdf]

```
                           1        10        20        30        40        50        60
A/Victoria/2570/2019(H1N1) MKAILVVMLYTFTTANADTLCIGYHANNSTDTVDTVLEKNVTVTHSVNLLEDKHNGKLCK
A/Astrakhan/3212/2020(H5N8) .MENIVLLLAIVSLVKSDQICIGYHANNSTEQVDTIMEKNVTVTHAQDILEKTHNGKLCD
mHAs                       MKAILVVMLYTFTTANADTLCIGYHANNSTDTVDTVLEKNVTVTHSVNLGSG........

                                    70        80        90       100       110       120
A/Victoria/2570/2019(H1N1) LRGVAPLHLGKCNIAGWILGNPECESLSTARSWSYIVETSNSDNGTCYPGDFINYEELRE
A/Astrakhan/3212/2020(H5N8) LNGVKPLILKDCSVAGWLLGNPMCDEFIRVPEWSYIVERANPANDLCYPGSLNDYEELKH
mHAs                       ............................................................

                                   130       140       150       160       170       180
A/Victoria/2570/2019(H1N1) QLSSVSSFERFEIFPKTSSWPNHDSDNGVTAACPHAGAKSFYKNLIWLVKKGKSYPKINQ
A/Astrakhan/3212/2020(H5N8) LLSRINHFEKILIIPK.SSWPNHETSLGVSAACPYQGAPSFFRNVVWLIKKNDAYPTIKI
mHAs                       ............................................................

                                   190       200       210       220       230       240
A/Victoria/2570/2019(H1N1) TYINDKGKEVLVLWGIHHPPTIADQQSLYQNADAYVFVGTSRYSKKFKPEIATRPKVRDQ
A/Astrakhan/3212/2020(H5N8) SYNNTNREDLLILWGIHHSNNAEEQTNLYKNPTTYISVGTSTLNQRLVPKIATRSQVNGQ
mHAs                       ............................................................

                                   250       260       270       280       290       300
A/Victoria/2570/2019(H1N1) EGRMNYYWTLVEPGDKITFEATGNLVAPRYAFTMERDAGSGIIISDTPVHDCNTTCQTPE
A/Astrakhan/3212/2020(H5N8) RGRMDFFWTILKPDDAIHFESNGNFIAPEYAYKIVKKGDSTIMKSGVEYGHCNTKCQTPV
mHAs                       ............................................................

                                   310       320       330       340       350
A/Victoria/2570/2019(H1N1) GAINTSLPFQNVHPITIGKCPKYVKSTKLRLATGLRNVPSIQSR...GLFGAIAGFIEGG
A/Astrakhan/3212/2020(H5N8) GAINSSMPFHNIHPLTIGECPKYVKSNKLVLATGLRNSPLREKRRKRGLFGAIAGFIEGG
mHAs                       ............................LRLATGLRNVPSIQSR...GLFGAIAGFIEGG

                           360       370       380       390       400       410
A/Victoria/2570/2019(H1N1) WTGMVDGWYGYHHQNEQGSGYAADLKSTQNAIDKITNKVNSVIEKMNTQFTAVGKEFNHL
A/Astrakhan/3212/2020(H5N8) WQGMVDGWYGYHHSNEQGSGYAADKESTQKAIDGVTNKVNSIIDKMNTQFEAVGREFNNL
mHAs                       WTGMVDGWYGYHHQNEQGSGYAADLKSTQNAIDKITNMVNSVIEKMGSG...........

                           420       430       440       450       460       470
A/Victoria/2570/2019(H1N1) EKRIENLNKKVDDGFLDIWTYNAELLVLLENERTLDYHDSNVKNLYEKVRNQLKNNAKEI
A/Astrakhan/3212/2020(H5N8) ERRIENLNKKMEDGFLDVWTYNAELLVLMENERTLDFHDSNVKNLYDKVRLQLRDNAKEL
mHAs                       ...................TDLAELLVLLLNERTLDYHDSNVKNLYEKVRNQLKNNAKEI

                           480       490       500       510       520       530
A/Victoria/2570/2019(H1N1) GNGCFEFYHKCDNTCMESVKNGTYDYPKYSEEAKLNREKIDGVKLDSTRIYQILAIYSTV
A/Astrakhan/3212/2020(H5N8) GNGCFEFYHKCDNECMESVRNGTYDYPQYSEEARLKREEISGVKLESIGTYQILSIYSTA
mHAs                       GNGCFEFYHKCDNTCMESVKNGTYDYPKYSEEAKLNREKID...................

                           540       550       560
A/Victoria/2570/2019(H1N1) ASSLVLVVSLGAISFWMCSNGSLQCRICI
A/Astrakhan/3212/2020(H5N8) ASSLALAIMMAGLSLWMCSNGSLQCRICI
mHAs                       .............................
```
